# Encapsulation of Micro- and Milli-Sized Particles with a Hollow-Type Spherical Bacterial Cellulose Gel via Particle-Preloaded Droplet Cultivation

**DOI:** 10.3390/ijms20194919

**Published:** 2019-10-04

**Authors:** Toru Hoshi, Masashige Suzuki, Mayu Ishikawa, Masahito Endo, Takao Aoyagi

**Affiliations:** 1 Department of Materials and Applied Chemistry, College of Science and Technology Nihon University, 1-8-14, Kandasurugadai, Chiyoda-ku, Tokyo 101-8308, Japan; cstms5113@gmail.com (M.S.); csmy15019@g.nihon-u.ac.jp (M.I.); aoyagi.takao@nihon-u.ac.jp (T.A.); 2 Graduate School of Science and Technology, Nihon University, 1-8-14, Kanda-Surugadai, Chiyoda-ku, Tokyo 101-8308, Japan; csma14028@g.nihon-u.ac.jp

**Keywords:** bacterial cellulose, hollow sphere, encapsulation, silicone oil

## Abstract

A hollow-type spherical bacterial cellulose (HSBC) gel prepared using conventional methods cannot load particles larger than the pore size of the cellulose nanofiber network of bacterial cellulose (BC) gelatinous membranes. In this study, we prepared a HSBC gel encapsulating target substances larger than the pore size of the BC gelatinous membranes using two encapsulating methods. The first method involved producing the BC gelatinous membrane on the surface of the core that was a spherical alginate gel with a diameter of 2 to 3 mm containing the target substances. With this method, the BC gelatinous membrane was biosynthesized using *Gluconacetobacter xylinus* at the interface between the cell suspension attached onto the alginate gel and the silicone oil. The second method involved producing the BC gel membrane on the interface between the silicone oil and cell suspension, as well as the spherical alginate gel with a diameter of about 1 mm containing target substances. After the BC gelatinous membrane was biosynthesized, an alginate gel was dissolved in a phosphate buffer to prepare an HSBC gel with the target substances. These encapsulated substances could neither pass through the BC gelatinous membrane of the HSBC gel nor leak from the interior space of the HSBC gel. These results suggest that the HSBC gel had a molecular sieving function. The HSBC gel walls prepared using these methods were observed to be uniform and would be useful for encapsulating bioactive molecules, such as immobilized enzymes in HSBC gel, which is expected to be used as a drug carrier.

## 1. Introduction

Since bacterial cellulose (BC) biosynthesized using *Acetobacter xylinum* was first discovered [1], it has been used in various practical applications [2,3,4,5]. BCs have good mechanical and physical properties, including high tensile strength, modulus, water-holding capacity, porosity, and crystallinity, as well as good biocompatibility [6]. Given these characteristics, BC gel has been studied as a drug carrier for drug delivery systems [7,8] and a scaffold for tissue engineering [9,10]. In these applications, shape control of the BC gel is a critical factor; as such, tubular and spherical BC gels have been reported [11,12]. Czaja et al. reported that *Acetobacter xylinum* produces isolated sphere-type cellulose under certain agitated culture conditions [13]. Putra et al. reported a simple technique to biosynthesize tubular BC gel with a desired length, inner diameter, and thickness [14]. Another example of BC gel shape control is the ear-shaped BC gel that was biosynthesized using a silicone mold for tissue engineering [15]. Since shape control of a BC gel via forming and machining after production is difficult, a cultivation method to produce a BC gel with the desired shape suitable for the purpose is required. We previously successfully prepared a hollow-type spherical bacterial cellulose (HSBC) gel [16]. The BC gelatinous membrane was biosynthesized using *Gluconacetobacter xylinus* at the interface between the cell suspension and silicon oil. HSBC gels were prepared using floating droplets of cell suspension in a mixture of two silicone oils with different densities. The gelatinous membrane of the HSBC gel was formed via the network structure of cellulose nanofibers with a diameter of approximately 30 nm. Various molecules that are smaller than the pore size of the cellulose nanofiber network can permeate the gelatinous membrane of the HSBC gel according to Fickian diffusion [16]. The gelatinous membrane of the HSBC gel functions as a barrier against larger molecules. Therefore, molecules larger than the pore size of the cellulose nanofiber network cannot be loaded into an HSBC gel. 

Encapsulation technology has been studied in various fields, such as pharmaceuticals, food science, paints, cosmetics, and adhesives. Specifically, the encapsulation of islet cells has received considerable attention, and the encapsulation would be effective for the treatment of type I diabetes. Lim and Sun demonstrated prolonged isograft islet survival using alginate-polylysine-polyethyleneimine microcapsules [17]. Post-transplantation, the encapsulated islets continued to live for up to three weeks without immunosuppression, compared with eight days for unencapsulated islets. O’Shea et al [18]. removed the polyethyleneimine component and used alginate as the outer layer of a microcapsule. These alginate-polylysine microencapsulated islets survived for the duration of the 365-day experiment in one of the five animals used. Encapsulation of islet cells with alginate demonstrated excellent results, but problems with biocompatibility were reported. Improvement of the biocompatibility of alginate microcapsules is necessary to reduce the impurities [19] and to increase the ratio of guluronic acid to mannuronic acid [20]. Alternative microencapsulated materials to alginate have been explored, including polyethylene glycol, poly (methyl methacrylate), agarose, chitosan, collagen, and gelatin [21,22,23]. A nanoporous thin-film cell encapsulation device made from poly(ε-caprolactone) (PCL) showed cell viability in allogeneic mouse models for up to 90 days [24].

Many types of natural and synthetic polymers have been investigated to find an optimal encapsulation material. Encapsulation uses a selectively permeable membrane that permits the passive diffusion of glucose, insulin, oxygen, carbon dioxide, and other nutrients, while preventing direct contact of the encapsulated cells with immune cells. The hydrodynamic diameters of globular proteins range between 1 and 10 nm; small molecules including organic metabolites are between 0.5 and 1 nm in diameter [25,26,27]. Thus, globular proteins and organic metabolites are expected to be able to pass through the BC membrane given the nanometer-sized pores of the HSBC gel. Our previous report demonstrated that fluorescein isothiocyanate (FITC) dextran (molecular weight (M.W.) 1 × 10 ^4^, hydrodynamic radius: approx. 2.3 nm [28]) was loaded inside the HSBC and released by diffusion. Immunological cells, such as macrophages and leukocytes, are 6–10 microns [29] in diameter, so they cannot pass through the BC membrane. HSBC gels, given their excellent mechanical properties, acid, and base resistance, and selective permeability by size exclusion, may be suitable for cell encapsulation.

**Figure 1:**
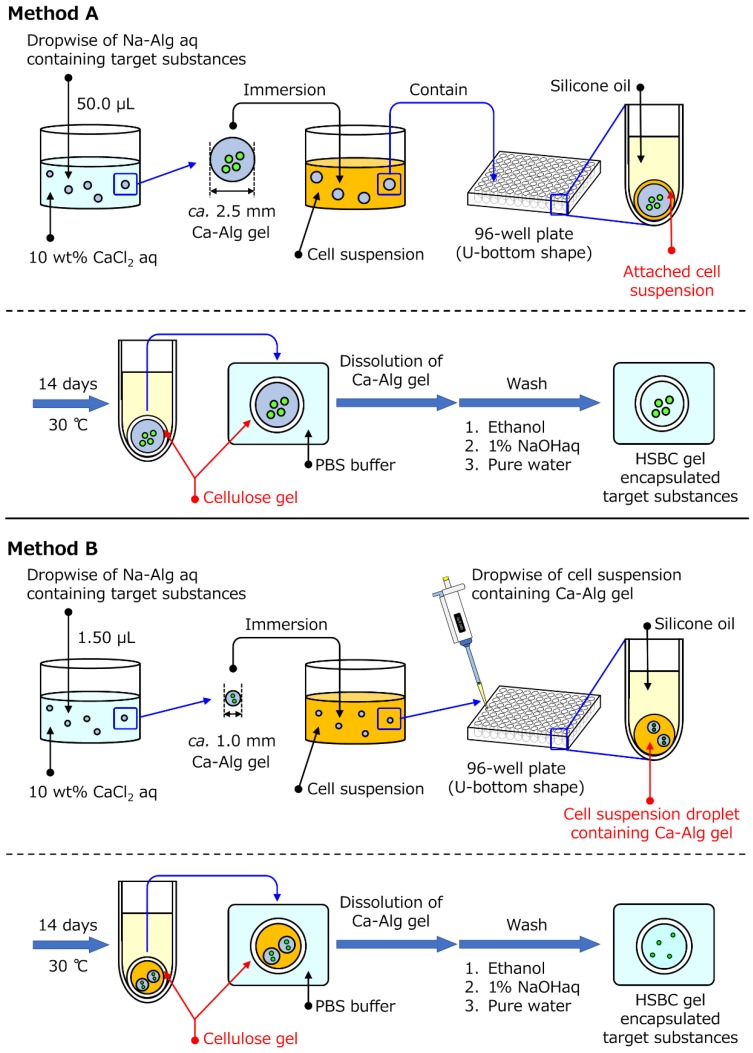
Schematic representation of methods for the production of the hollow-type spherical bacterial cellulose (HSBC) gel encapsulating millimeter- and micrometer-sized particles. (Method A) HSBC gel encapsulating millimeter-sized particles. (Method B) HSBC gel encapsulating micrometer-sized particles.

**Figure 2:**
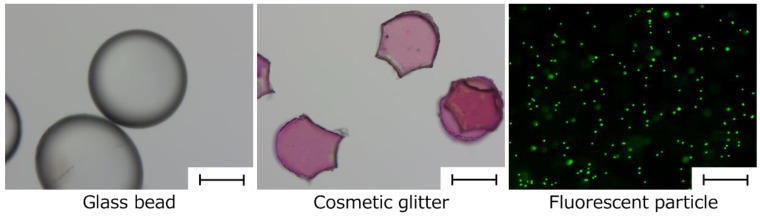
Photographs of target substances used for encapsulation. Scale bar: 200 µm.

In the present study aiming at encapsulating cells, we prepared an HSBC gel encapsulating substances larger than the pore size of BC gelatinous membranes. Two encapsulating methods were used. The first method involved producing the BC gelatinous membrane on the surface of a spherical alginate gel with a diameter of 2 to 3 mm containing target substances to be encapsulated in the HSBC gel (Figure 1, Method A). The second method involved producing the BC gel membrane on the interface between a silicone oil and a cell suspension, as well as a spherical alginate gel with a diameter of about 1 mm containing target substances (Figure 1, Method B). After production of the BC gelatinous membrane, the substance-encapsulated HSBC gel was obtained by dissolving the alginate gel. Figure 2 shows the target substances used for encapsulation. Glass beads with diameters of 0.3–0.5 mm, cosmetic glitter with diameters of 0.3–0.5 mm, and fluorescent particles with diameters of 5.0–5.9 µm were used as targets for encapsulation within the HSBC gel. Glass beads are large and have a large specific gravity. Cosmetic glitter is large and has an anisotropic shape. These substances were used in method A to encapsulate large particles. The fluorescent particles were approximately the same size as the cell and were used to examine the permeability of the BC gelatinous membrane in method B.

## 2. Results

### 2.1. Preparation of the HSBC Gel Encapsulating Large Particles Using Method A 

**Figure 3:**
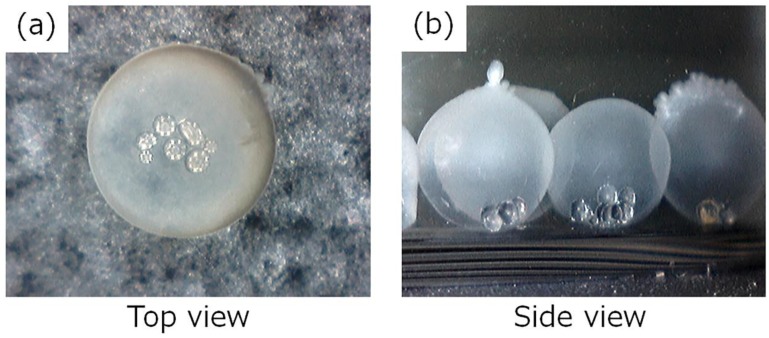
Photographs of HSBC-gel-encapsulated glass beads: (**a**) top view and (**b**) side view.

Using the calcium alginate (Ca-Alg) gel containing glass beads as model substances, we attempted encapsulation with the BC gelatinous membrane because glass beads that were much larger than the pore size of cellulose nanofiber network could not permeate the BC gelatinous membrane. Figure 3 depicts the microscopic view of the HSBC gel; the presence of glass beads in the interior space of the HSBC gel was confirmed. The glass beads could move within the HSBC gel, but some beads stuck to the inner surface of the HSBC gel. Glass beads with a higher specific gravity sank in the sodium alginate aqueous solution (Na-Alg aq), and parts of the glass beads were exposed on the surface of the Ca-Alg gel. As described below, the viscosity of the Na-Alg solution seemed to be important for the sticking of the beads. When the BC gel membrane was produced, the glass beads were immobilized by the interaction via hydrogen bonds and the entanglement effect between the exposed part of the glass beads and the cellulose fibers. As shown in Figure 3, a few beads were observed and the number of encapsulated glass beads within the HSBC gel was the same as the number of beads in the Ca-Alg gel. If the Ca-Alg gel can be loaded with more beads, many glass beads could be encapsulated within the HSBC gel.

When we used a high concentration of the Na-Alg aq, the obtained Ca-Alg gels were more stable with less swelling. However, the more difficult aspect of the viscous Na-Alg solution was micropipette suction, and the suction including large particles, such as glass beads and cosmetic glitter, was difficult. In contrast, with the low Na-Alg aq concentration, the micropipette suction was easy, but the obtained Ca-Alg gel swelled with water and the medium. The size of the HSBC gel was controlled by the particle size of the Ca-Alg gel. A change in the size of the Ca-Alg gel due to swelling was undesirable. For Method A, Ca-Alg gel prepared with 1.0 wt% Na-Alg aq hardly swelled in the medium; therefore, 1.0 wt% Na-Alg aq was more appropriate.

**Figure 4:**
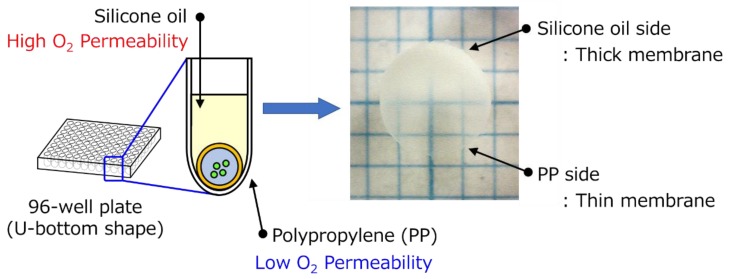
Non-uniformity of the gelatinous membrane thickness of the HSBC gel produced using method A.

The HSBC gel prepared using the Ca-Alg gel had a non-uniform membrane thickness (Figure 4). In the thick membrane region, *G. xylinus* was cultured at the interface between the cell suspension attached onto the Ca-Alg gel and the silicone oil. Where the membrane was thin, the Ca-Alg gel was attached to the bottom part of the 96-well plate composed of polypropylene (PP). Two reasons explain the difference in the membrane thickness. The first is the difference in oxygen permeability between silicone oil and the PP. *G. xylinus*, which are aerobic bacteria, require oxygen to produce cellulose. Thus, cellulose was likely biosynthesized on the side of the silicone oil that had a higher oxygen permeability than PP; therefore, the BC gelatinous membrane was thicker as a result. The second was the difference in the direction of the growth of the BC gelatinous membrane. In our previous study [16], we reported that the membrane thickness gradually increased from the surface to the internal direction of the cell suspension droplet. At the interface between the cell suspension attached onto the Ca-Alg gel and the 96-well plate, the BC membrane could not grow in either direction, resulting in the formation of a thin gelatinous membrane.

**Figure 5:**
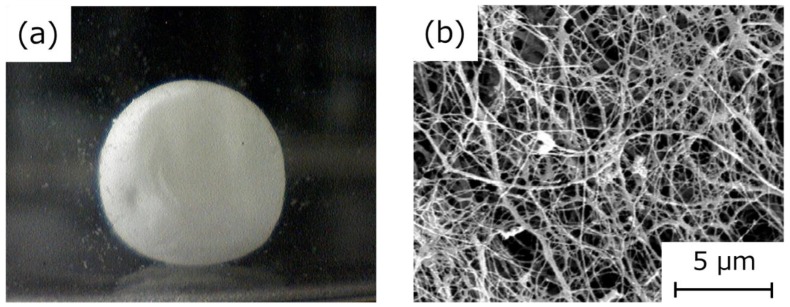
(**a**) Photograph of the HSBC aerogel. (**b**) Scanning electron microscopy (SEM) image of HSBC aerogel.

The HSBC gel encapsulating the glass beads was cleaned with phosphate-buffered saline (PBS) immediately after culturing. After removing the silicone oil using ethanol, the HSBC gel encapsulating the glass beads was dried using the supercritical drying method. Observation of the external appearance of this HSBC gel revealed that the BC gelatinous membrane was formed on the entire surface of the sphere (Figure 5a). Scanning electron microscopy (SEM) observation of the HSBC aerogel indicated a network structure of cellulose nanofibers with a diameter of about 30 nm (Figure 5b). These results mean that the BC gel film was produced at the interface between the cell suspension attached onto the Ca-Alg gel and the silicone oil or the 96-well plate.

### 2.2. Optimization of the Dissolving Process of the Ca-Alg Gel and Silicone Oil

**Figure 6:**
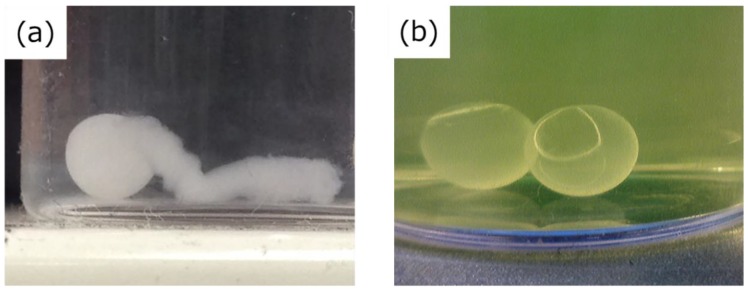
(**a**) HSBC gel perforated by swollen alginate gel in PBS. (**b**) Perforated HSBC gel after removal of the Ca-Alg gel. This perforated HSBC gel was immersed in fluorescein isothiocyanate (FITC)-dextran aqueous solution so that the pores were easy to observe.

The silicon oil used in this study was soluble in ethanol. When the Ca-Alg gel was dissolved using PBS [30] after washing with ethanol, a hole unexpectedly appeared in the BC gelatinous membrane of the HSBC gel due to swelling of the Ca-Alg gel (Figure 6a). After the cultivation, the obtained gel, which was Ca-Alg gel covered with the BC gelatinous membrane, was immediately immersed in ethanol to remove the adhered silicone oil prior to dissolution of the Ca-Alg gel. As a result, Ca-Alg gel that existed came into contact with the ethanol that passed through the BC gelatinous membrane. The Ca-Alg gel shrunk in ethanol and stiffened due to shrinking. Therefore, the part of the Ca-Alg gel in contact with the permeated ethanol shrunk and hardened. These shrunken parts of the Ca-Alg gel did not dissolve with PBS, so these parts acted as physically cross-linked points. In the replacement of Ca^2+^ via immersion in PBS for the removal of alginate, this shrunken Ca-Alg gel did not dissolve in PBS completely and was more swollen compared with before its immersion in ethanol. As a result, perforated HSBC gels were obtained (Figure 6b).

In contrast, when the obtained gel was immediately immersed in PBS, the PBS gradually passed through the BC gelatinous membrane and dissolved the Ca-Alg gel without rapid swelling, and no holes formed during the dissolving process (Figure 5a). The formation of a spherical-shaped BC gelatinous membrane on the entire surface was observed, and the BC gelatinous membrane was not perforated due to the swelling of Ca-Alg gel during the dissolving process. Also, the silicone oil adhered onto the surface of the obtained gels did not dissolve in PBS but floated out to the PBS. Therefore, the silicone oil could be separated from the gels.

### 2.3. Preparation of the HSBC Gel Encapsulating Millimeter-Size Particles with a Controlled Shape Using Method A

**Figure 7:**
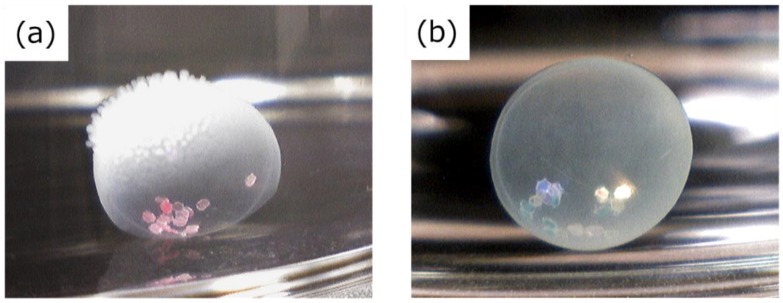
Photographs of HSBC gel encapsulating cosmetic glitter. (**a**) Encapsulation by non-uniform and (**b**) uniform BC gelatinous membranes.

As described above, we first attempted to prepare the HSBC gel with a uniform membrane thickness via reducing the number of cultivation days. However, the BC gelatinous membrane of the HSBC gel was perforated during the dissolving process until the sixth day of incubation. HSBC gels cultured for more than seven days were not perforated and differed in film thickness but could encapsulate the cosmetic glitter (Figure 7a). This result suggests that the BC gelatinous membrane was weak. The production of BC nanofibers by *G. xylinus* was enhanced to produce a BC gelatinous membrane sufficiently strong enough to encapsulate some substances in the inner space. Therefore, we used the cell suspension with a three-fold glucose concentration to increase the production of BC nanofibers and turned the Ca-Alg gel upside down on day 5 of the cultivation period to produce a uniform membrane thickness. After seven days of incubation, we successfully obtained a more spherical HSBC gel that encapsulated cosmetic glitter (Figure 7b).

### 2.4. Preparation of the HSBC Gel Encapsulating Fluorescent Particles Using Method B

**Figure 8:**
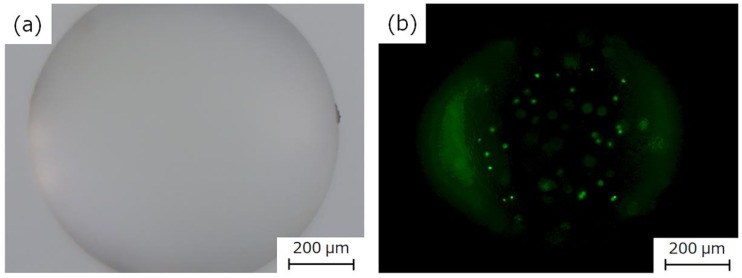
Photographs of Ca-Alg gel including fluorescent particles: (**a**) optical image and (**b**) fluorescent image.

We prepared 2 mL of 0.5 wt% Na-Alg aq with 200 µL of 0.1% (*w/v*) fluorescent particles. Figure 8 depicts the Ca-Alg gel encapsulating the fluorescent particles with a diameter of 1.0 mm that were prepared by dropping 1.50 µL of this aqueous solution on 10 wt% CaCl_2_ aq. Fluorescent particles were present inside the Ca-Alg gel and they did not leak out, as shown in Figure 8. In method B, the cell suspensions containing the small Ca-Alg gel were cultured in silicone oil. The size of the HSBC gel produced with this method was almost the same as the cell suspension droplet. The size of the HSBC gel was not affected even if the Ca-Alg gel swelled. To prepare the small-sized Ca-Alg gel, minute amounts of Na-Alg aq must be accurately suctioned using a micropipette. The low concentration and low viscosity Na-Alg aq was suitable for this purpose. However, when the amount of suspended substances in Na-Alg aq increased, no spherical Ca-Alg gel was obtained at low Na-Alg concentrations. Na-Alg aq droplets did not undergo gelation because the amount of Na-Alg was too low. Thus, 0.5 wt% Na-Alg aq was found to be appropriate.

**Figure 9:**
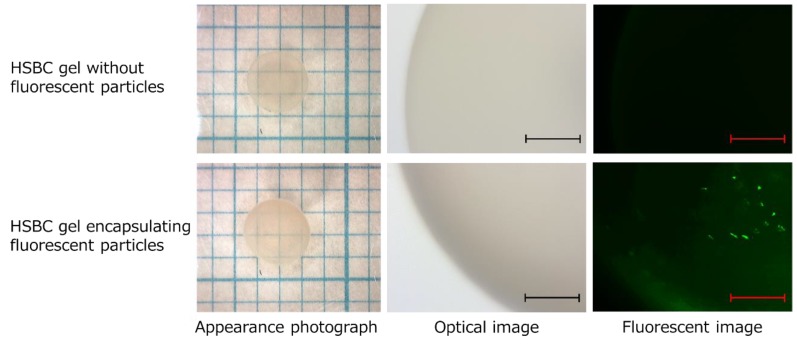
Photographs of HSBC gel without fluorescent particles and HSBC gel encapsulating fluorescent particles. Scale bar: 300 µm.

The cell suspension droplets, including the above-mentioned small Ca-Alg gel containing fluorescent particles, were prepared by dropping them into the silicone oil and incubating for 14 days. The HSBC gels encapsulating fluorescent particles were obtained by processing them as follows: the Ca-Alg gel of the obtained gels was dissolved with PBS followed by removal of the silicon oil with ethanol. Regardless of the presence or absence of the small Ca-Alg gel, the diameter of the obtained HSBC gel was 2.9 mm (radius (r) = 0.145 cm) and almost consistent with the 10-µL droplet (droplet volume = 10 µL = 1.0 × 10 ^−3^ cm ^3^ = 4πr ^3^/3; r = 0.134 cm) (Figure 9).

**Figure 10:**
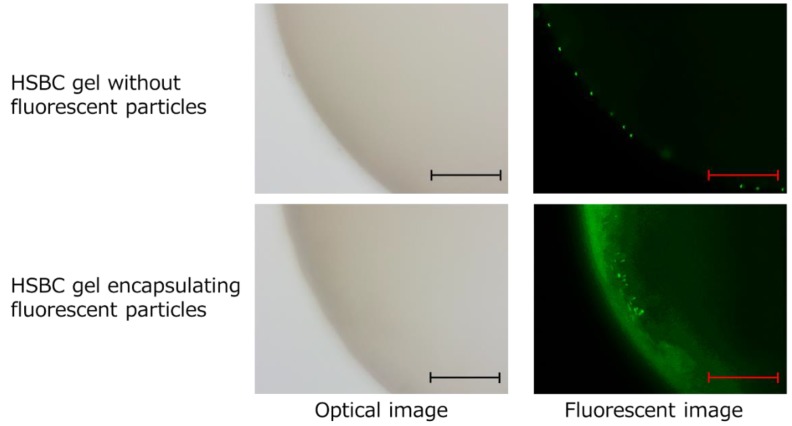
Photographs of the blank HSBC gel immersed in fluorescent particles aqueous solution and HSBC gel encapsulating fluorescent particles. Scale bar: 300 µm.

The fluorescent-particle-loaded HSBC gel was observed with a fluorescence microscope to investigate whether the particles would leak out (Figure 10). As a control experiment, HSBC gel was immersed in aqueous solution containing 0.01% (*w/v*) fluorescent particles for 24 h. As a result, fluorescent particles were observed only on the outer surface of the HSBC gel, not the inner space. Fluorescent particles that were larger than the pore size of the network structure of the BC gelatinous membrane adhered to the outer surface of the HSBC gel, and could not penetrate into the inner space of the HSBC gel.

The HSBC gel encapsulating the fluorescent particles was stored in Milli-Q water for at least two weeks and was observed using fluorescence microscopy. Fluorescent particles were only observed in the interior space of the HSBC gels and the particles were observed to be adsorbed on the inner surface of the HSBC gel. Fluorescent particles could not pass through the BC gelatinous membrane of the HSBC gel; thus, they remained in the interior space of the HSBC gel. These results indicate that encapsulated substances that were larger than the pore size of the cellulose nanofiber network structure did not leak out from the interior space of the HSBC gel.

## 3. Discussions

In this study, we successfully encapsulated substances larger than the pore size of the cellulose nanofiber network structure with a BC gelatinous membrane via an HSBC gel preparation method using a Ca-Alg gel. Encapsulated substances could not pass through the BC gelatinous membrane of the HSBC gel and did not leak from the interior space of the HSBC gel. The BC gelatinous membrane prevented the invasion of substances larger than the pore size into the HSBC gel. In contrast, molecules smaller than the pore size could pass through the BC gelatinous membrane, so these molecules were loaded into the interior space of the HSBC gel. These results show that the HSBC gel demonstrated a molecular sieving function.

Encapsulation experiments in this study were performed at room temperature and at 30 °C, and we used compounds with low toxicity. Thus, encapsulation of biocatalysts, enzymes, and cells with HSBC gel without denaturation and devitalization was anticipated. Globular proteins and organic metabolites that are between 0.5 and 10 nm in diameter are expected to be able to pass through the BC membrane with nanometer-sized pores of the HSBC gel according to Fickian diffusion. The BC network is known to be impenetrable by cells due to the small size of the pores [6,31]. For example, enzyme-immobilized beads encapsulated in HSBC gel may react with permeated substrates, such as globular proteins and organic metabolites. These enzyme-immobilized beads may not be attacked by immune cells that are several microns across and cannot pass through the BC membrane. Thus, as the BC gel membrane is a functional membrane with a molecular sieve effect and biocompatibility, enzyme-immobilized bead-encapsulated HSBC gel may provide a small reaction field in an in vivo environment.

Many researchers have shown that BC gel supports the adhesion and proliferation of different cell types [32,33,34,35]. The degradation of cellulose in animal and human tissues is limited, if it occurs at all, due to the absence of hydrolases that attack the β (1,4) linkage. Therefore, encapsulation of cells with HSBC gel may allow for long-term culturing of multiple types of cells in the same environment for the isolation of cells via the BC gel membrane. Helenius et al. demonstrated the in vivo biocompatibility of BC gel [36]. BC gel was implanted subcutaneously in rats for several weeks. The implants were evaluated for chronic inflammation, foreign body responses, cell ingrowth, and angiogenesis. No macroscopic and microscopic signs of inflammation around the implants were observed, and no fibrotic capsule or giant cells were present. Fibroblasts infiltrated the BC, which was well integrated into the host tissue, and did not elicit any chronic inflammatory reactions. In the islet encapsulation with alginate hydrogels, transplantation of the capsules including cells leads to a host response that depends on multiple factors, such as the cells and materials used, the transplant site, and so on [37]. Shortly after transplantation into tissues, the host response to transplantation and the material may include an inflammatory response with nearby blood vessels. However, because BC gel has the abovementioned excellent biocompatibility, HSBC gels encapsulating the islet cells may be transplanted without an inflammatory response. Encapsulation with HSBC gel has the potential to be used as a scaffold in tissue engineering.

## 4. Materials and Methods

### 4.1. Materials

Hestrin–Schramm’s (HS) medium [38] was used for incubation of the bacterial strain. Standard HS medium consisted of a mixture of 3.0 g D-glucose (Kanto Chemical Co. Inc., Chou-ku, Tokyo, Japan), 0.5 g mannitol (Kanto Chemical Co. Inc.), 0.5 g peptone (HIPOLYPEPTONE^TM^, Nihon Pharmaceutical Co. Ltd., Chou-ku, Tokyo, Japan), 0.5 g Bacto^TM^ yeast extract (BD Biosciences, Franklin Lakes, New Jersey, USA), and 0.1 g magnesium sulfate heptahydrate (MgSO_4_∙7H_2_O; Kanto Chemical Co. Inc.) in 100 mL Milli-Q water (Merck Millipore, Burlington, Massachusetts, USA). The density of the HS medium at 30 °C was measured to be 1.02 g/cm ^3^ using a Baume hydrometer (Nihon keiryouki kougyou Co. Ltd., Chiyoda-ku, Tokyo, Japan). Other reagents were purchased from Kanto Chemical Co. Inc., and used as-received. Silicone oils (KF-56A: 0.995 g/cm ^3^, 15 mm/s^2^, ethanol-soluble oil) were obtained from Shin-Etsu Chemical Co., Ltd (Chiyoda-ku, Tokyo, Japan). Fluorescent particles (Spectro^TM^ FITC particles, 5.0–5.9 µm, 0.1% (*w/v*) PBS, pH 7.4) were purchased from Spherotech Inc. (Lake Forest, Illinois, USA), and used as-received.

### 4.2. Preparation of Spherical Alginate Gel Including Millimeter-Sized Particles

Spherical alginate gels (Ca-Alg gel) including the target substances (glass beads or cosmetic glitter) were prepared by dropping 50 µL of 1 wt% Na-Alg aqueous solution (Na-Alg aq) including these substances into a 10 wt% calcium chloride aqueous solution (CaCl_2_ aq). To drop 1 wt% Na-Alg aq including glass beads or cosmetic glitter, an Eppendorf pipette tip (100 µL capacity, Eppendorf Co. Ltd., Chiyoda-ku, Tokyo, Japan) cut 1 cm from the leading edge was used. Obtained Ca-Alg gels were washed by immersing in Milli-Q water. 

### 4.3. Preparation of the Alginate Gel Including Fluorescent Particles

The Ca-Alg gels with a diameter of 0.9 mm including the fluorescent particles were prepared using the method described below. We prepared 2 mL of 0.5 wt% Na-Alg aqueous solution containing 200 µL of the solution containing fluorescent particles. We dropped 1.50 µL of this Na-Alg aq into the 10 wt% CaCl_2_ aq, and the obtained Ca-Alg gels were stored in a cool, dark place before use. They were washed with Milli-Q water and used for the incorporation experiment using a fluorescence microscope (ZEISS Axio Scope.A1, Axiocam 105 color, Carl Zeiss Microscopy GmbH, Jena, Germany).

### 4.4. Preparation of the Hollow-Type Spherical BC Gels Encapsulated Target Substances

Figure 1 showed the preparation method to get the target substances encapsulated by HSBC gel. Method A was used to prepare the HSBC-gel-encapsulated millimeter-sized particles, such as glass beads and cosmetic glitter as model substances, and method B was used for the encapsulation of micrometer-sized particles, such as fluorescent particles.

The HS medium was sterilized via autoclaving, and then *G. xylinus* (IFO13772) was cultured in the HS medium at 30 °C for 3 days. The prepared spherical Ca-Alg gels that included the target substances were immersed in the cultured cell suspension and inoculated with *G. xylinus* for one day at 30 °C. In method A, spherical Ca-Alg gels, where the cell suspension remained at the gel surface, were immersed in each well of a 96-well plate (Caplugs Evergreen, Buffalo, New York, item: 290-8353-03R) filled with silicone oil. In method B, a 10-µL cell suspension with small Ca-Alg gels including micrometer-sized particles was dropped in each well filled with silicone oil. While maintaining these states, *G. xylinus* in the cell suspension was cultured for a specified period at 30 °C. This 96-well plate had a U-shaped bottom and was composed of PP. In both methods A and B, the BC gelatinous membrane was produced on the interface of the cell suspension and the silicon oil. After 2 weeks, the resulting gel was immersed in phosphate buffered saline (PBS) to dissolve the Ca-Alg gel. After removing the Ca-Alg gel, the remaining silicone oil on the HSBC gel was removed by washing with ethanol. The substance-encapsulated HSBC gel thus produced was purified by soaking it in a large amount of distilled water for 1 day, followed by washing in a 1% (*w/v*) aqueous solution of NaOH at room temperature for 1 additional day to remove the bacterial cell debris and alkali-soluble components completely. Then, it was washed several times with a large amount of distilled water and stored in distilled water at room temperature.

### 4.5. Preparation of HSBC Aerogel Using Supercritical CO_2_

Water-swollen HSBC gel was placed in a large quantity of methanol, washed thoroughly, and the swelling solvent was completely changed from water to methanol. The gel was dried using a supercritical CO_2_ (scCO_2_) technique without disintegrating its microstructure [39]. The drying was conducted under conditions of 40 °C, 20 MPa, and CO_2_ flow rate 2.0 mL/min for 5 h. The drying apparatus consisted of a CO_2_ delivery pump (SCF-Get, JASCO Co., Hachioji-shi, Tokyo, Japan), 50 mL pressure vessel, a gas pressure regulator (SCF-Bpg, JASCO Co., Japan), and a constant-temperature water bath (BK33, Yamato Scientific Co. Ltd., Chuo-ku, Tokyo, Japan).

### 4.6. Characterization of HSBC Gels

The microstructure of HSBC aerogels was observed using a field-emission scanning electron microscope (FE-SEM: S-4500, Hitachi High-Technologies Corporation, Minato-ku, Tokyo, Japan) with an acceleration voltage of 10 kV. For the pretreatment prior to FE-SEM observation, a deposition of Pt-Pd was performed using ion sputtering (E-1010, Hitachi High-Technologies Corporation).
